# Parenting Style and Emotional Well-Being Among Adolescents: The Role of Basic Psychological Needs Satisfaction and Frustration

**DOI:** 10.3389/fpsyg.2022.901646

**Published:** 2022-06-15

**Authors:** Fitri Ariyanti Abidin, Whisnu Yudiana, Syipa Husni Fadilah

**Affiliations:** ^1^Faculty of Psychology, Center for Innovation and Psychological Research, Universitas Padjadjaran, Sumedang, Indonesia; ^2^Department of General and Experimental Psychology, Faculty of Psychology, Universitas Padjadjaran, Sumedang, Indonesia

**Keywords:** parenting style, emotional well-being, adolescents, basic psychological need satisfaction, basic psychological need frustration

## Abstract

The research examined the relationship between supportive parenting styles (warmth, structure, and autonomy support) and emotional well-being and whether they are mediated by basic psychological need satisfaction. It also explores thwarting parenting styles (rejection, chaos, and coercion) that may be associated with emotional ill-being, mediated by basic psychological needs frustration. This study involved 394 Indonesian adolescents aged 11–15 years old (49.5% boys, 50.5% girls) as the participants. We employed the structural equation model (SEM) analysis to evaluate the hypotheses. The research found that basic psychological needs satisfaction fully mediated the relationship between supportive parenting styles and emotional well-being; basic psychological needs frustration fully mediated the relationship between thwarting parenting styles and emotional ill-being (Chi-Square = 434.39; df = 220; *p* = 0.000; RMSEA = 0.05; CFI = 0.91; GFI = 0.91; SRMR = 0.05). Interestingly, the findings indicate that the thwarting parenting style positively influences basic psychological needs satisfaction. The research concludes that supportive parenting enhances the well-being of adolescents by satisfying their basic psychological needs. However, thwarting parental behaviors did not forestall the satisfaction of needs. The way Indonesian adolescents perceived the thwarting parenting style was discussed.

## Introduction

Emotional well-being is essential in adolescents experiencing multiple transitions (physiological, psychosocial, academic, and social) and shifts in family dynamics. Those transitions could bring pressures that may induce stress, leading to mental health problems (Johnson and Greenberg, 2013). Kim-Cohen et al. (2003) further suggest that the onsets of mental health problems usually appear in late childhood and adolescence. Therefore, emotional well-being is crucial for adolescents to have flourishing mental health. Adolescents with emotional well-being tend to be physically and mentally healthy. They can avoid abusing the internet and drugs (Saha et al., 2014), gain higher academic achievement, possess good intrapersonal and interpersonal skills, and apply a better coping strategy when facing life problems (Shek and Lin, 2014). Those positive traits may continue in the future, as positive well-being experienced in adolescents tends to remain in adulthood.

One theory that concerns individual well-being is The Self-Determination Theory (SDT), stating that individuals’ three basic psychological needs, namely the need for relatedness, competence, and autonomy, could be satisfied or thwarted. The satisfaction of relatedness needs will make individuals feel connected in a quality relationship with others, the satisfaction of competence needs will make individuals feel productive, and the satisfaction of autonomy needs will encourage individuals to control their choice of behavior (Ryan and Deci, 2017). The satisfaction of all the needs leads to the achievement of emotional well-being. Many studies reported a relationship between positive outcomes and satisfaction, in which psychological needs are associated with vital and energetic feelings (Chen et al., 2015b; Pinquart, 2016), life satisfaction (DeHaan et al., 2016), and more significant positive effect (Sheldon and Bettencourt, 2002).

Contrarily, frustration in relatedness needs might encourage individuals to feel a sense of rejection and alienation. Unfulfillment of the competence needs tends to lead to feelings of inferiority and helplessness, while the frustration of autonomy needs creates a sense of coerciveness to individuals (Ryan and Deci, 2017). Vansteenkiste and Ryan (2013) suggest that frustration of the needs will result in emotional ill-being. Furthermore, the effect of basic need’s frustration on mental health problems includes symptoms of eating disorders (Boone et al., 2014), anxiety and somatization (Cordeiro et al., 2016), and sleep deprivation (Campbell et al., 2017).

Initially, needs satisfaction was identified as a uni-dimensional construct ranging from low to high (Sheldon et al., 1996; Deci et al., 2001; Jang et al., 2009). However, further studies found that although low needs satisfaction is associated with lower well-being, it is not directly associated with ill-being (Ryan et al., 1995; Adie et al., 2008; Quested and Duda, 2010). Therefore, Bartholomew et al. (2011) propose a dual-process model to distinguish need (dis)satisfaction and need frustration. In other words, basic psychological needs satisfaction and frustration are defined as multidimensional. These notions are supported by Vansteenkiste and Ryan (2013), reporting the relationship between psychological need satisfaction and frustration is asymmetrical, whereby low need satisfaction is not a condition for the presence of need frustration. On the contrary, a high need frustration implies a low need satisfaction.

To date, SDT researchers agree that needs satisfaction and needs frustration are different constructs, as they have different antecedents and outcomes (Chen et al., 2015a; Haerens et al., 2015; Jang et al., 2016; Bartholomew et al., 2018). Chen et al. (2015a) further developed a measurement of BPNSFS that views needs satisfaction and needs frustration as different constructs. Research on BPNSFS in various populations and languages corroborates the notion that the needs satisfaction and needs frustration are distinct constructs; thus, their measurement and interpretation should be differentiated. To conclude, as the basic psychological need satisfaction and frustration are viewed as different constructs, to find that individuals have high satisfaction and high frustration at the same time is possible (Cordeiro et al., 2016; Nishimura and Suzuki, 2016; Valle et al., 2018; Rodrigues et al., 2019; Liga et al., 2020).

Whether the basic psychological needs in adolescents are fulfilled or thwarted, strongly associated with parents, the social context has been known to have a significant impact on the development, functioning, and well-being (Costa et al., 2019) as well as the ill-being of adolescents. Several studies have documented the impact of parenting on adolescents’ well-being. Niemiec et al. (2006), for example, reported that support from parents affects adolescents’ well-being, while Wimsatt et al. (2013) suggest that there is a relationship between parental involvement and positive affect. Whereas, studies also found that parenting can affect adolescents’ ill-being. For instance, King et al. (2018) found that poor monitoring/supervision, inconsistent discipline, and corporal punishment are related to depressive symptoms. In addition, a higher risk for suicidal adolescents increases in conjunction with parenting behaviors.

Parents’ influence on their children could be understood from three aspects: parenting goal (the goals parents promote), parenting style (emotional climate within the family), and parenting practice (behaviors that parents do to reach the parenting goals) (Darling and Steinberg, 1993). Parenting style and parenting practice are often used interchangeably. Nevertheless, the two terms should be distinguished as the former focuses on how parents do the parenting, while the latter focuses more on concrete behaviors parents do (Power, 2013). Since the 1940s, more attention has been given to research on parenting styles than parenting practices, which cannot predict individual differences in children’s social/emotional development (Orlansky, 1949).

Parenting style can be investigated by two major approaches: the dimensional approach studying each parenting dimension independently, and the typological approach combining specific dimensions of parenting into parenting styles (Power, 2013). Based on an extensive literature review on parenting dimensions, Skinner et al. (2005) propose six-core parenting dimensions: warmth, structure, autonomy support, rejection, chaos, and coercion. The warmth dimension emphasizes feelings of affection, unconditional love, and emotional support; the structure dimension prioritizes clear explanation and proper limitation, while the autonomy-supportive dimension is indicated by giving opportunities to express feelings and opinions comfortably. On the contrary, rejection is usually indicated by negative and hostile expressions shown by parents, chaos is shown by a lack of consistency in rules and parental behaviors, and coercion is characterized by strict parental control.

As for parenting typologies, Baumrind’s typological approach has been considered one of the prominent theories combining two orthogonal dimensions of parenting: responsiveness and demandingness (Darling and Steinberg, 1993; Martinez et al., 2019; Martinez-Escudero et al., 2020). Demandingness refers to setting up a reasonable demand accompanied by monitoring and disciplinary efforts (Baumrind, 1991; Garcia et al., 2020). Responsiveness is indicated by intentional action to encourage individuality, self-regulation, and self-assertion while considering the child’s personality (Baumrind, 1991; Garcia et al., 2020). The combination of high demandingness and high responsiveness forms an authoritative parenting style, a combination of high demandingness and low responsiveness form an authoritarian parenting style, while a combination of high responsiveness and low demandingness forms a permissive parenting style (Baumrind, 2013).

Skinner et al. (2005) suggests that different dimensions could be aggregated. However, unlike Baumrind combining two dimensions into a particular parenting style, Skinner proposes three dimensions aggregated into supportive parenting (warmth, structure, and autonomy support) and unsupportive parenting (rejection, chaos, and coercion). This categorization firstly was proposed based on the SDT framework, which posits that children have three basic psychological needs: related, competence, and autonomous. The empirical finding further supports this categorization, which showed high intercorrelation among warmth, structure, and autonomy support, and among rejection, chaos, and coercion. Skinner’s and Baumrind’s parenting style categorization might sound similar, especially between authoritative and supportive parenting styles. Nevertheless, this study uses Skinners’ proposition, as it separates autonomy support from structure/demandingness (Skinner et al., 2005). The recent studies under the umbrella of SDT corroborated Skinners’ categorization. Aligns with SDT tenets about basic psychological needs; warmth, structure, and autonomy support are labeled need-supportive parenting, while rejection, chaos, and coercion are labeled need-thwarting parenting (Costa et al., 2019; Soenens et al., 2019).

Regarding the impact of parenting styles on children’s outcomes, several factors should be taken into consideration. Cultural context (Garcia et al., 2020; Gimenez-Serrano et al., 2021; Gimenez-Serrano et al., 2022), and neighborhood (Sandoval-Obando et al., 2022) play an important role in the association between parenting style and children’s outcomes. Numerous research under Baumrind’s parenting style framework found that in the Western population, authoritative parenting is well-known as the best parenting style (Devi and Uma, 2013; Sahithya et al., 2019). However, the best parenting style is not always the same in the different populations and cultures. In the Asian and African contexts, the authoritarian parenting style is considered better to socialize children (White et al., 2009). Therefore, the recommendation to apply a particular parenting style should consider the cultural context (Pinquart and Kauser, 2018).

As for Skinner’s categorization, the reports on the impact of supportive and thwarting parenting styles in a different culture have also been documented. Some studies suggest that the parenting style has a similar impact despite the cultural setting. In Western countries, supportive parenting promotes the child’s positive outcomes by enhancing children’s well-being, whereas thwarting parenting is associated with internalizing or externalizing problems (Joussemet et al., 2008; Soenens et al., 2017). A similar finding was reported in non-Western populations, such as Taiwanese populations. The study found the effect of need-supportive parenting on intrapersonal and interpersonal adaptation was mediated by need satisfaction (Wu et al., 2015). These findings might be expected as the SDT suggests the notion applied universally. Nevertheless, given the complex characteristics of the cultural context and the more research under SDT conducted in various cultures, different findings might be revealed.

The relationship between the parenting dimensions and satisfaction of basic psychological needs has been documented in several studies. Skinner et al. (2005) and Joussemet et al. (2008) found that parental warmth plays a vital role in adolescents’ relatedness, while parental autonomy support fosters adolescents’ autonomy. This finding is corroborated by Flamm and Grolnick (2013), who found that the parental structure shapes experiences of competence. Likewise, the satisfaction of the three needs is substantially influenced by parental structure and autonomy support (Abidin et al., 2019). Furthermore, Kocayoruk (2012), investigating the relationship between parenting behavior, basic needs, and adolescents’ well-being, revealed that parental support indirectly influences adolescents’ subjective well-being, mediated by basic psychological needs. Costa et al. (2015) reported that the satisfaction of basic psychological needs mediated the relationship between perceptions of psychological control and internalizing distress. Costa et al. (2019) further explained that basic psychological needs were mediating parenting style and outcomes in Italian adolescents. The findings indicate that supportive parenting practice (autonomy support, structure, and warmth) increases need fulfillment and adjustment, while thwarting parenting practice (psychological control, chaos, and rejection) has the opposite effect on need fulfilment and adjustment. Furthermore, the relationship between parenting and adolescents’ adjustment are mediated by the basic psychological needs, which are need for autonomy, competence, and relatedness.

**FIGURE 1:**
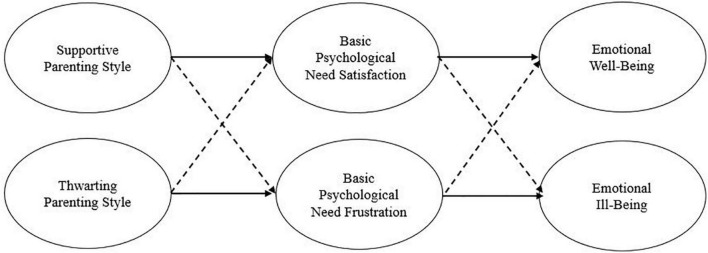
The conceptual model of relationships between parenting style, basic psychological need, and well-being. The solid line indicates a positive relation. The dashed line indicates a negative relation.

Several researchers reported the relationship between parenting behavior, basic psychological needs satisfaction, well-being, and ill-being. However, studies that examine whether basic psychological needs frustration mediate thwarting parenting styles and emotional ill-being are relatively scarce. According to SDT, basic psychological needs frustration is a different construct from basic psychological needs satisfaction; thus, research focusing on examining basic psychological needs frustration as a mediating variable is crucial. The current study aims to examine the relationship between supportive parenting styles and emotional well-being, and whether the basic psychological need satisfaction mediates the relationship. Moreover, to fill the gaps of the previous research, this research also examines the relationship between thwarting parenting styles and emotional ill-being and whether basic psychological need frustration mediates the relationship (Figure 1).

Based on previous research, we expect that a supportive parenting style (warmth, structure, autonomy support) will positively influence basic psychological needs satisfaction, which will result in higher emotional well-being; whereas thwarting parenting style (rejection, chaos, and coercion) will positively influence basic psychological needs frustration which will result in higher emotional ill-being.

## Materials and Methods

### Participants

The participants of this study were 394 students from three senior high schools, of which 49.5% were males and 50.5% were females. The age of the participants ranged from 11 to 15 years old (M = 12.98, SD = 0.77) and the proportion of the participants, based on their grades at school, were: 64.23% 7th-year students, 31.97% 8th-year students, and 3.81% 9th-year students.

### Procedures

The Research Ethics Committee of Universitas Padjadjaran has given this study their approval (No. 357/UN6.KEP/EC/2018). Prior to signing the consent form, the participants have received information about the aim, data collection procedure, and data protection of the study. The participants, guided by the research assistants, completed the paper-and-pencil questionnaires for 15–30 min in the regular class periods.

### Measures

#### Parenting Style

The Parents as Social Context Questionnaire (PSCQ)- Adolescent Report in Indonesian version (Abidin et al., 2019) was employed to measure the parenting style. The instrument consists of six parental dimensions, namely warmth (e.g., “My parents think I am special”), rejection (e.g., “Nothing I do is good enough for my parents”), structure (e.g., “When I want to do something, my parents show me how”), chaos (e.g., “My parents punish me for no reason”), autonomy support (e.g., “My parents trust me”), and coercion (e.g., “My parents are always telling me what to do”), with four items for each dimension. Participants answered the questionnaire on 4-point Likert scales (1 = not at all true to 4 = very true). The research reported that the PSCQ had good internal consistency reliability, as the scores for warmth, rejection, structure, chaos, autonomy support, and coercion are 0.83, 0.80, 0.79, 0.74, 0.70, and 0.66, respectively.

#### Basic Psychological Needs

The Indonesian version of the Basic Psychological Needs Satisfaction and Frustration Scale (BPNSF), adapted from Chen et al. (2015a) by Abidin et al. (2021) was employed to measure basic psychological needs. Generally, the scale consists of six subscales measuring satisfaction of autonomy (e.g., “I feel a sense of choice and freedom in the things I undertake”), relatedness (e.g., “I feel connected with people who care for me and for whom I care),” and competence (e.g., “I feel capable at what I do”) and the frustration of autonomy (e.g., “I feel pressured to do too many things”), relatedness (e.g., “I feel the relationship I have are just superficial”), and competence (e.g., I feel insecure about my abilities”), with four items for each subscale. However, in the Indonesian version, two items from the autonomy frustration (“Most of the things I do feel like I have to” and “My daily activities feel like a chain of obligation”) were excluded from the instruments due to the negative factor loading. Participants rated the instrument on the 5-point Likert scale (1, not true at all; 5, completely true). The internal consistency for the basic psychological needs satisfaction and frustration subscales were acceptable, with α = 0.791 and α = 0.709, respectively.

#### Emotional Well-Being and Emotional Ill-Being

Subjective feelings of well-being and ill-being were measured by the Scale of Positive and Negative Experience (SPANE; Diener et al., 2010). SPANE-P (Positive Emotional Experience; e.g., “joyful”) and SPANE-N (Negative Emotional Experience; e.g., “angry”) were measured by six items in the instrument. Items were scored on a 5-point Likert scale (1 = very rarely or never, 5 = very often or always). This scale has achieved internal consistency in the Indonesian sample with α = 0.77 for the SPANE-P and α = 0.75 for the SPANE-N.

### Analytic Plan

A preliminary exploration related to the missing value was conducted using Little’s MCAR test. Descriptive statistics, the means and standard deviations for each variable were described. Then, Pearson’s product-moment correlations were selected to examine the relationship between variables and the existence of multicollinearity. Next, serial structural model equation (SEM) examinations were administered to investigate the mediation role of basic psychological needs in the relation between parenting style and emotional well-being. Basically, this research consisted of two outcome variables (emotional well-being and emotional ill-being), two predictor variables (supportive parenting style and thwarting parenting style), and two mediator variables (basic psychological needs satisfaction and basic psychological needs frustration). Thus, the evaluation of mediation variables using SEM examinations followed Holmbeck (1997) causal approach.

First, the analysis was conducted to gain an adequate overall model fit when the predictor variables are regressed on the outcome variables (Model 1). The path coefficients are expected to be significant. Then, the analysis was followed by assessing the overall model fit when predictor variables are regressed on the mediator variables, and simultaneously the outcome variables are regressed on the mediator variables (Model 2). The model fit must be good, and all path coefficients must be significant. In the final step, the addition of the paths on Model 2, between predictor and outcome variables is not constrained to zero and has to be significant. The mediational effect exists when the addition paths between predictors and outcomes in the unconstrained model do not significantly increase the fit compared to the constrained model.

Several models fit indices were used in this study, including chi-square (χ^2^), root mean square error of approximation (RMSEA), an absolute fit index GFI, and two other common fit indices: the comparative fit index (CFI), and the standardized root mean square residual (SRMSR). The cut-off point for fit indices followed these criteria: the values of GFI and CFI should be at least 0.90, the RMSEA and SRMSR values should be less than 0.08 (Hair et al., 2014). Statistical Product and Service Solution (SPSS) 22.0 for Mac was utilized to perform descriptive statistical analysis and correlation analysis. Meanwhile, the SEM was administered using the Lavaan package on R programming (Rosseel, 2012).

## Results

### Preliminary Analysis and Descriptive Statistics

The examination of missing values indicated approximately 21 (5.32%) missing values were found from the participants. The maximum missing value of each participant is 5.60%. A Little’s MCAR test found that the missing values were random, as χ^2^ = 605.49 (df = 592; *p* = 0.34); thus, we employed the expectation-maximization method for missing value imputation (Hair et al., 2014). The means, standard deviations, and bivariate correlations between the observed variables for each construct are shown in Table 1.

**TABLE 1:** Means, standard deviations, and correlations between measurement variables.

Variable	1	2	3	4	5	6	7	8	9	10	11	12	13	14
1. Emotion well-being														
2. Emotion ill-being	−0.28**													
3. Relatedness satisfaction	0.25**	−0.20**												
4. Competence satisfaction	0.24**	−0.23**	0.46**											
5. Autonomy satisfaction	0.16**	−0.10*	0.40**	0.37**										
6. Relatedness frustration	−0.16**	0.23**	−0.17**	–0.03	0.05									
7. Competence frustration	−0.22**	0.38**	−0.21**	−0.33**	–0.06	0.44**								
8. Autonomy frustration	−0.25**	0.28**	−0.13*	−0.21**	–0.02	0.36**	0.49**							
9. Warmth	0.28**	−0.22**	0.45**	0.36**	0.25**	−0.19**	−0.24**	−0.23**						
10. Structure	0.20**	−0.16**	0.45**	0.39**	0.30**	−0.15**	−0.25**	−0.21**	0.55**					
11. Autonomy support	0.24**	−0.24**	0.52**	0.38**	0.38**	−0.23**	−0.28**	−0.27**	0.64**	0.58**				
12. Rejection	−0.15**	0.23**	−0.17**	−0.14**	0.00	0.45**	0.38**	0.25**	−0.31**	−0.20**	−0.30**			
13. Chaos	−0.14**	0.15**	–0.03	−0.11*	0.05	0.33**	0.39**	0.23**	−0.22**	−0.11*	−0.17**	0.49**		
14. Coercion	–0.02	–0.01	0.06	0.08	0.10*	0.21**	0.13*	0.00	0.03	0.11*	–0.02	0.35**	0.42**	
Mean	3.94	2.44	3.79	3.55	3.36	2.21	2.32	2.52	3.44	3.29	3.39	1.96	2.33	2.4
SD	0.59	0.63	0.73	0.71	0.64	0.73	0.67	0.97	0.49	0.49	0.43	0.56	0.59	0.56

*p < 0.05; **p < 0.01.

As expected, emotional well-being correlates negatively with emotional ill-being. Emotional well-being has a positive correlation with three basic needs satisfaction and three dimensions comprising supportive parenting style, while correlating negatively with three basic psychological needs frustration and three dimensions comprising thwarting parenting style. Such a paradoxical pattern identified in emotional ill-being, as it has a negative correlation with three basic needs satisfaction and three dimensions comprising supportive parenting style, but correlates positively with three basic psychological needs frustration and three dimensions comprising thwarting parenting. Unexpectedly, coercion as one of the dimensions of thwarting parenting style does not significantly correlate with emotional well-being and ill-being, even though it has a significant correlation with other thwarting parenting dimensions (i.e., rejection and chaos).

### Mediational Analysis

**FIGURE 2:**
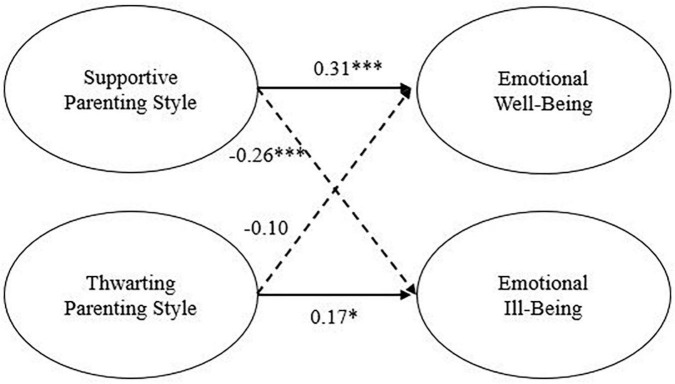
Standardized structural equation of Model 1. Solid lines indicated positive relations, and dashed lines indicated negative relations. *p < 0.05, **p < 0.01, ***p < 0.001.

**FIGURE 3:**
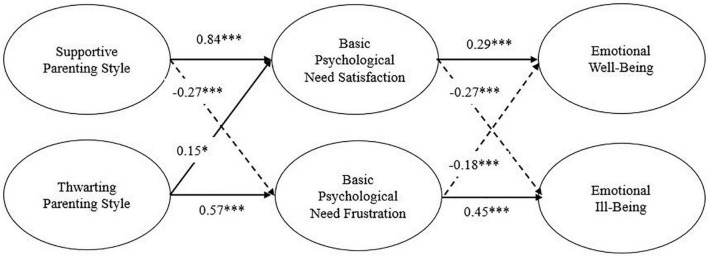
Standardized structural equation of Model 2. Solid lines indicated positive relations, and dashed lines indicated negative relations. *p < 0.05, **p < 0.01, ***p < 0.001.

The analyses of mediational effect consisted of three steps of structural equation models. First, Model 1, analyzing the direct effect of the supportive parenting style and thwarting parenting style on emotional well-being and emotional ill-being, showed a good model fit (see Table 2) and significant path coefficients. Specifically, Figure 2 shows that the supportive parenting style had a significant positive direct effect on emotional well-being and a significant negative direct effect on emotional ill-being. Conversely, thwarting parenting style only had a significant positive direct effect on emotional ill-being. Next, the mediation variables (basic psychological needs satisfaction and basic psychological needs frustration) were included in Model 2, in which the goodness of fit for the proposed model was satisfactory according to all fit indices (see Table 2). Figure 3 shows that all estimated parameters were significant and of acceptable magnitude. Supportive parenting style demonstrated a significant and high positive direct relationship with basic psychological need satisfaction (γ = 0.84, *p* < 0.001) and a positive indirect relationship with emotional well-being (γ = 0.24), mediated by basic psychological need satisfaction. While thwarting parenting style had a positive and direct relation with basic psychological need frustration (γ = 0.57, *p* < 0.001) and a positive indirect relationship with emotional ill-being (γ = 0.26), mediated by basic psychological need frustration. In addition, the basic psychological need had a direct and positive association with emotional well-being (β = 0.29, *p* < 0.001). Similarly, a positive relationship was found between basic psychological needs frustration, and emotional ill-being (β = 0.45, *p* < 0.001). Finally, negative associations were found between supportive parenting style and basic psychological need frustration, basic psychological need satisfaction and emotional ill-being, and between psychological need frustration and emotional well-being. A different result is found on the relationship between thwarting parenting style and basic psychological need satisfaction, which has no significant association. This model accounted for 20% of the variance in emotional well-being and 29% in emotional well-being.

**TABLE 2:** Fit Indexes of Model 1, 2 and 3.

	χ^2^	df	χ^2^/df	RMSEA	CFI	GFI	SRMR
Model 1	230.07	113.00	2.04	0.05	0.93	0.94	0.06
Model 2	434.39	220.00	1.97	0.05	0.91	0.91	0.05
Model 3	432.31	216.00	2.00	0.05	0.91	0.91	0.06

*χ^2^, Chi-square; RMSEA, root mean square error of approximation residual; CFI, comparative fit index; GFI, goodness of fit index; SRMR, Standardized root mean square.*

**FIGURE 4:**
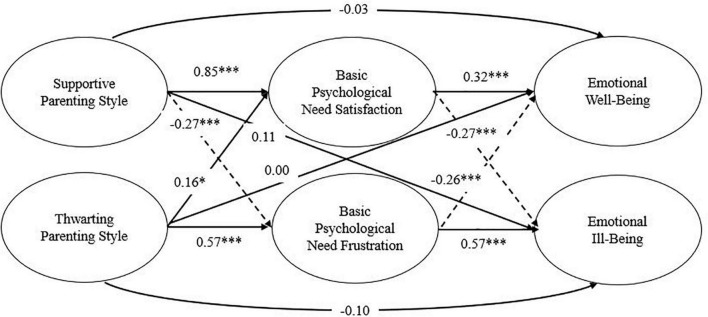
Standardized structural equation of Model 3. Solid lines indicated positive relations, and dashed lines indicated negative relations. *p < 0.05, **p < 0.01, ***p < 0.001.

The analysis of Model 3 was almost similar to Model 2, except the model also examined the significance of direct paths between predictor variables and outcome variables. The results showed that the direct effect from predictor variables to outcome variables was not significant (Figure 4) and the overall model fit of Model 3 was not different from Model 2 (Table 2). The results concluded that the basic psychological needs satisfaction and basic psychological needs frustration fully mediated the relationship between parenting style and emotional well-being.

## Discussion

The study examines the relationship between supportive parenting styles and emotional well-being, and whether basic psychological need satisfaction mediates the relationship. It also examines the relationship between thwarting parenting styles and emotional ill-being, and whether basic psychological needs frustration mediates the relationship. Congruent with the hypotheses, the findings showed that supportive parenting style positively influences basic psychological needs satisfaction, which results in higher emotional well-being. In a similar vein, thwarting parenting style positively influences basic psychological needs frustration, resulting in higher emotional ill-being.

The findings are in line with Kocayoruk (2012); Ahmad et al. (2013), and Costa et al. (2019), reporting that supportive parenting predicts the psychological needs fulfillment and positive outcomes, while psychological needs frustration and negative outcomes are reflected from thwarting parental practices. Their research also found that psychological needs mediate the relationship between supportive parenting and positive outcomes, and the relationship between thwarting parenting and negative outcomes. Ryan and Deci (2017) found that thwarting parental behaviors hindered the need for competence and induced inferior feelings that make adolescents prone to subjective distress, negative feelings, and affection.

The key finding of this study supports the SDT claim that basic psychological needs represent nourishment necessary for well-being and that children’s frustration is likely to induce negative behaviors (Deci and Ryan, 2000; Ryan and Deci, 2000). In SDT, supportive parenting could serve as a unique predictor for need satisfaction, while thwarting parenting would work to predict need frustration (Bartholomew et al., 2011; Vansteenkiste and Ryan, 2013; Costa et al., 2015).

Different from most previous studies, this study revealed that thwarting parenting style positively predicted the basic psychological needs satisfaction. The correlational result showed that parental coercion might be a contributing factor to this finding. Even though numerous evidence shows that controlling parenting has effects on children’s negative outcomes, some cross-cultural studies identified less negative or even non-existent effects of controlling parenting in Eastern Asian samples (e.g., Iyengar and Lepper, 1999). These findings might reflect the less pressuring and more favorable meaning of controlling parenting in East Asian culture (Park and Kim, 2004; Grusec, 2012). The case illustrated by Chao (1994) shows that Asian children are likely to view parenting control as a concern or engagement. On the contrary, such a parenting style will hold less positive meaning in an individualistic culture, as it is considered a mere channel of parents’ anger and rejection.

As parenting is heavily driven by culture (Bornstein, 2012), this finding accentuates that the culture of the West and the East differs. The former views coercion as a thwarting parenting style where parents shoulder responsibility for their children’s wrong doing (Senese et al., 2012). Parenting goals in the Western cultures characterize this conceptualization to educate children to become outspoken, self-reliant, capable, and self-determined (Rubin et al., 2008). In Eastern culture, on the other hand, parenting is often associated with high expectations and control from parents (Chao and Aque, 2009). This belief stems from the hierarchical structure that demands younger individuals respect and regards their elders highly. Indonesian parents also instill a similar value which requires children to submit to their parents’ guidance and hold any questions, particularly about religion and cultural issues.

This study strongly represents a comprehensive examination of parenting style, basiychological needs, well-being, and ill-being, using the SDT framework. The findings particularly add to a growing literature on the basic psychological need theory. However, the present findings might have a limitation as it uses correlational data that does not reflect cause and effect. To address this issue, future research should apply experimental or longitudinal methods. Furthermore, this study may reflect the findings in Indonesian adolescents. However, it might not reveal the specific family condition, which might differ among populations, as we didn’t collect the data related to the family background. To gain a deeper understanding of the topic, future research should take these variables into account.

### Implications for Practices, Application, and Policy

The findings of this study have an important implication for future practice. It confirmed the significance of supportive parenting in the fulfillment of three basic psychological needs satisfaction, which eventually promotes adolescents’ well-being. Various interventions to provide knowledge and skills for parents to practice warmth, structure, and autonomy support are essential. Further research should be undertaken to explore the concept of thwarting parenting, especially coercion.

## Data Availability Statement

The datasets presented in this study can be found in online repositories. The names of the repository/repositories and accession number(s) can be found below: https://osf.io/698ja.

## Ethics Statement

The studies involving human participants were reviewed and approved by the Research Ethics Committee of Universitas Padjadjaran. Written informed consent to participate in this study was provided by the participants’ legal guardian/next of kin.

## Author Contributions

FA contributed to conception and design of the study. SF organized the database. WY performed the statistical analysis. FA and WY wrote the first draft of the manuscript. All authors wrote sections of the manuscript, contributed to manuscript revision, read, and approved the submitted version.

## Conflict of Interest

The authors declare that the research was conducted in the absence of any commercial or financial relationships that could be construed as a potential conflict of interest.

## Publisher’s Note

All claims expressed in this article are solely those of the authors and do not necessarily represent those of their affiliated organizations, or those of the publisher, the editors and the reviewers. Any product that may be evaluated in this article, or claim that may be made by its manufacturer, is not guaranteed or endorsed by the publisher.
